# St. Luke’s

**Published:** 1874-11

**Authors:** 


***St. Luke's Hospital.*** Surgical Service. Dr. OWENS.

A German laborer, twenty-two years of age, had a part of his left foot crushed by railroad accident. The great toe and its metatarsal bone were removed, together with the second toe, and the head of its metatarsal bone. After the wound began to granulate, thin grafts of skin were placed upon it at five various spots. The reparative process was remarkably hastened near and around these grafts as centres, which speedily spread out and united with the borders of the wound already healed.

